# Image-based Classification of Tumor Type and Growth Rate using Machine Learning: a preclinical study

**DOI:** 10.1038/s41598-019-48738-5

**Published:** 2019-08-29

**Authors:** Tien T. Tang, Janice A. Zawaski, Kathleen N. Francis, Amina A. Qutub, M. Waleed Gaber

**Affiliations:** 1 Department of Bioengineering, Rice University, 6500 Main Street, Suite 1030, Houston, TX 77030 USA; 2 Department of Pediatrics, Hematology-Oncology Section, Dan L. Duncan Cancer Center, Baylor College of Medicine, 1102 Bates Street, Suite 200, Houston, TX 77030 USA

**Keywords:** Paediatric cancer, Diagnostic markers, CNS cancer, Biomedical engineering

## Abstract

Medical images such as magnetic resonance (MR) imaging provide valuable information for cancer detection, diagnosis, and prognosis. In addition to the anatomical information these images provide, machine learning can identify texture features from these images to further personalize treatment. This study aims to evaluate the use of texture features derived from T_1_-weighted post contrast scans to classify different types of brain tumors and predict tumor growth rate in a preclinical mouse model. To optimize prediction models this study uses varying gray-level co-occurrence matrix (GLCM) sizes, tumor region selection and different machine learning models. Using a random forest classification model with a GLCM of size 512 resulted in 92%, 91%, and 92% specificity, and 89%, 85%, and 73% sensitivity for GL261 (mouse glioma), U87 (human glioma) and Daoy (human medulloblastoma), respectively. A tenfold cross-validation of the classifier resulted in 84% accuracy when using the entire tumor volume for feature extraction and 74% accuracy for the central tumor region. A two-layer feedforward neural network using the same features is able to predict tumor growth with 16% mean squared error. Broadly applicable, these predictive models can use standard medical images to classify tumor type and predict tumor growth, with model performance, varying as a function of GLCM size, tumor region, and tumor type.

## Introduction

Brain tumors are a common occurring cancer in the pediatric population, with approximately 24,000 new cases each year that are highly aggressive and generally have poor treatment outcomes, as demonstrated by a 5 year survival rate of 33.2%^1,2^. Management of these tumors usually includes intracranial surgery, radiation therapy and chemotherapy with treatment plans tailored to the diagnosed tumor type. Diagnosis of a brain tumor is made from a biopsy in combination with molecular tests on the resected tumor tissue. However for some patients, biopsy or surgical resection is not possible therefore diagnosis relies solely on medical images^1^. While biopsies are the gold-standard in diagnosis, they come with limitations such as surgical risks, limited spatial and temporal resolution, and subjectivity of immunohistochemical scoring^3,4^. Current advances in imaging technology and analysis has made it possible to establish tumor radiographic phenotype or radio-phenotype, offering an additional source of information to complement biopsies and an alternative diagnostic tool when biopsies are not possible^5–8^.

The prognoses for gliomas are extremely poor compared to medulloblastomas for the current standard of care, which includes surgical resection, radiation therapy and/or chemotherapy, in which 5 year survival is 15–35% compared to 70%, respectively^9,10^. For tumors such as medulloblastoma that are more responsive to treatment, it may not be necessary for young patients to undergo the inherently risky surgical resection. Furthermore, the ability to predict tumor growth can help clinicians make informed decisions in cases where treatment needs to be delayed. For children under the age of three, radiation therapy is delayed due to long-term side effects of radiation on the developing brain. Therefore, establishing at tumor’s growth rate can identify critical treatment time points^11,12^.

Currently in the clinic, imaging is primarily used for anatomical information such as assessing tumor volume and location, determining feasibility of surgical resection, and assessing response to treatment. Additional information can be extracted from these images using radiomics by mining the images for quantitative image features that are not intuitively observable, such as variance in neighboring pixel values^13,14^. Recent studies have found that these features can be informative of the tumor’s underlying molecular processes when integrated with machine learning techniques to provide valuable diagnostic, prognostic and predictive information^15–22^. In this work, we focused on texture features, derived from the gray level co-occurrence matrix (GLCM) which are commonly used in many different texture analysis. The GLCM is a square matrix that captures the frequency in which a combination of gray scale intensities occur with the dimensions determined by the number of gray levels^23^. Features derived from this matrix are informative of the spatial relationships between grayscale intensities such as amount of variation, disorder, or contrast within an image^23^. These features, however, can be sensitive to image processing which include acquisition, reconstruction protocols, and inter-scanner variability^24–28^. Independent of these systemic variations, the values of these features can also be affected by the GLCM size or the number of gray levels which is determined a priori to feature extraction. For a given image the number of gray levels (pixel intensity) is dependent on the bit depth of that image; with an 8 bit image having 256 possible values or 16 bit image having 65,536 possible values. For a higher bit image it is not practical to construct a 65,536 by 65,536 GLCM therefore the image is often rescaled to a more manageable bit size which can ultimately affect the values of the texture features derived from the matrix. There have been few studies on the impact of GLCM size on the image’s features values even though these features are commonly used in radiomic studies and are included in image analysis software tools^29–31^. Preclinical studies can be valuable to investigate the influence GLCM size has on classifier performance where imaging parameters can be controlled and confounding factors can be eliminated.

The aim of this work is to classify brain tumor type and predict tumor growth rate using texture features from T_1_-weighted post contrast MR scans in a preclinical model. Tumor regions were segmented using in-house software with GLCMs constructed for a single tumor slice or entire tumor volume. We investigated the sensitivity of texture features values to different GLCM sizes and how this affects the performance of different classifiers. Our preclinical model also allowed for the opportunity to systematically follow the growth of these tumors. To further explore the potential application of texture features derived from diagnostics images we used these features to predict tumor growth rate. Using, a shallow neural network, image features were used to predict the αβ value of an exponential function, a simple growth model that assumes that growth is proportional to the cell population, where the α and β values describes the initial volume and the growth rate respectively. Using machine learning we assess whether radiomics approaches have the potential to classify tumor type and predict tumor growth rate noninvasively by allowing clinicians to make better informed treatment decisions using standard medical images.

## Results

**Figure 1:**
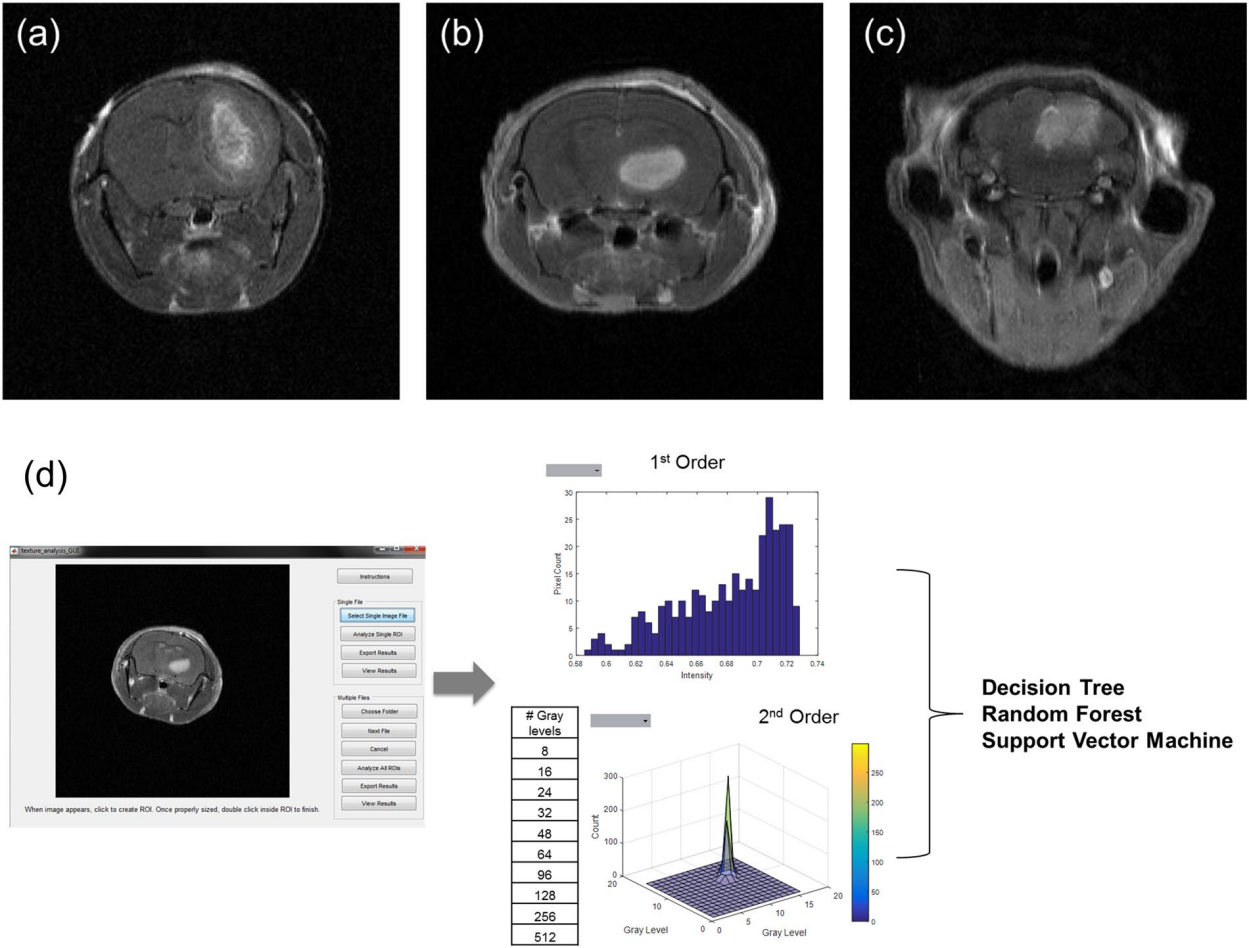
T1-weighted post contrast MR scan. (**a**) GL261, mouse glioma, (**b**) U87, human glioma, (**c**) Daoy, human medulloblastoma. (**d**) Schematic of workflow for feature extraction. Images are imported into a custom program for visualization and segmentation of the tumor region. The selected region used to extract 1^st^ and 2^nd^ order image features. These features are then used to construct the three different supervised classifiers: Decision Tree, Random Forest, and Support Vector Machine.

To construct our classification and prediction models, texture features were first extracted from the tumor region using in-house MATLAB program for three different types of tumors: GL261 (mouse glioma), U87 (human glioma) and Daoy (human medulloblastoma). The program consists of a graphical user interface (GUI) which allow users to import in DICOM files from T_1_-weighted post contrast scans either in batch or as a single image slice and perform manual segmentation of the region of interest (ROI). Image features were extracted from three different tumor regions (central, middle and edge) as well as the average of the entire tumor. Once segmentation is completed, the program calculates first and second order image features. First order features are derived from the grayscale intensity histogram of the selected ROI. Second order features are derived from ten different GLCM sizes. The extracted image features from the different GLCM sizes for each tumor type are used as inputs to train the three classifiers and tumor growth prediction models (Fig. 1).

### Tumor type classification

**Figure 2:**
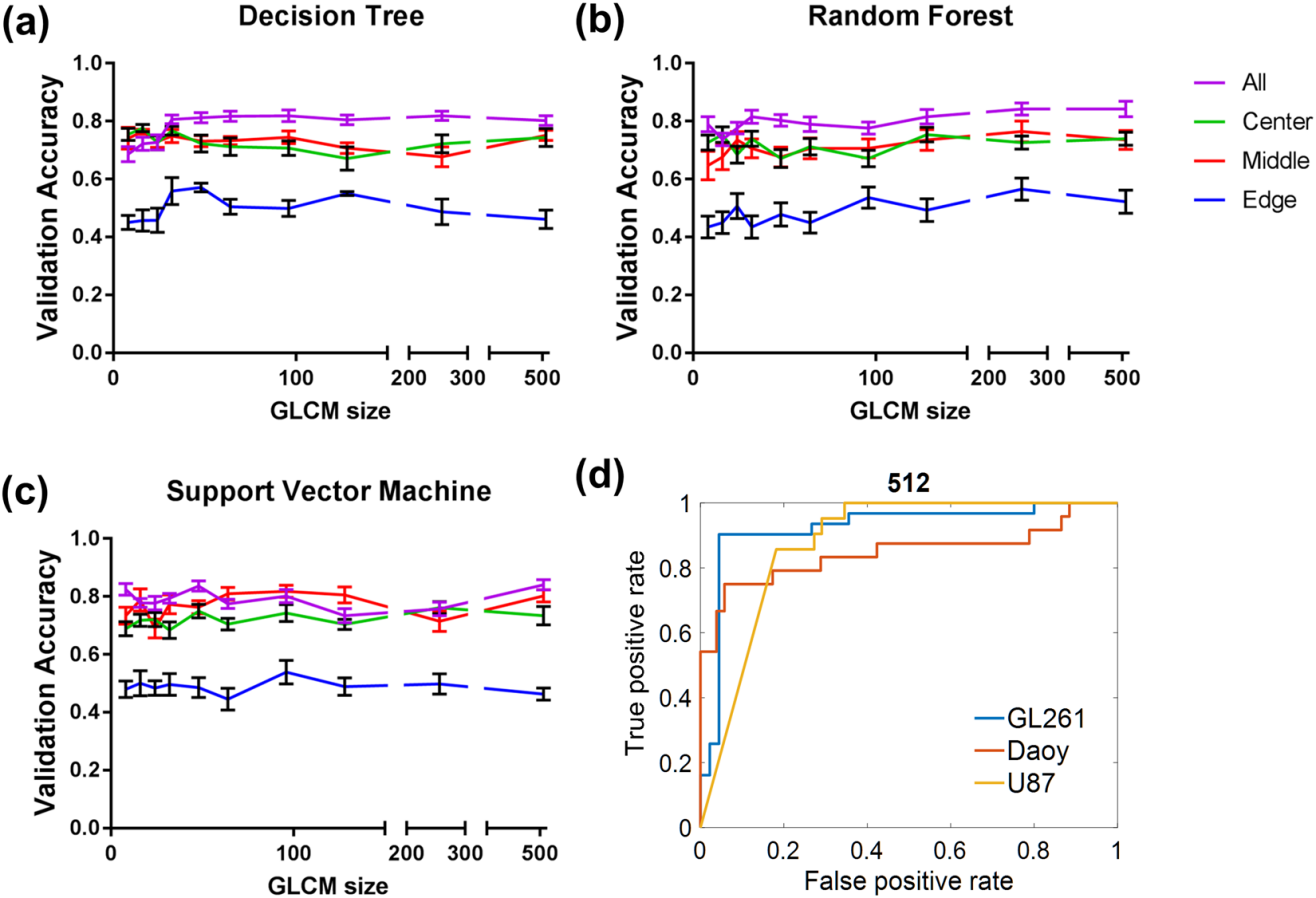
Validation accuracy of classifier across tumor regions and GLCM size. Model performance: (**a**) Decision Tree, (**b**) Random Forest and (**c**) Support Vector Machine, was consistently poor at the edge of the tumor and high with the entire tumor volume. Each data point represents a different matrix size. Data is shown as mean ± standard deviation. (**d**) Receiver operator curve of the best performing model, 512 gray levels with random forest.

Overall, the three different classification models, decision tree, random forest and support vector machine, had similar performances. Figure 2 shows the validation accuracy of the different GLCM sizes for each classifier and tumor region. We observed an overall trend of increasing validation accuracy with the number of gray levels or GLCM size, however it was not a proportional increase and varied for different classification models. The only instance in which increasing the GLCM size directly increased validation accuracy was when the entire tumor volume was used with a decision tree model (Fig. 2a). The selection of the tumor region used for GLCM construction also impacted the classifier’s accuracy. Using the entire tumor volume resulted in higher accuracy compared to a single image slice. The edge of the tumor was the least predictive while the central and middle regions were comparable (Fig. 2).

The best performing classification model was using random forest with 512 gray levels, equivalent to a GLCM size of 512, which achieved 84% validation accuracy. With the same model using the central, middle and edge tumor regions the validation accuracies were 74%, 74% and 52% respectively. The model resulted in an area under the curve (AUC) = 0.92, 0.88 and 0.85 for GL261, U87 and Daoy respectively (Fig. 2). While there was high specificity and sensitivity for both GL261 and U87 tumor classifications, sensitivity was only 0.77 for Daoy tumors (Tables 1–3). Even though the GLCM size of 256 had similar accuracy, the classifier performance was not as uniform for all tumor types as with the GLCM size 512. Therefore all subsequent analysis were performed on features extracted from the GLCM size of 512. We’ve also identified the four texture features dependent on GLCM size: autocorrelation, cluster prominence, sum of square and sum variance (p ≤ 0.01–0.0001) (see Supplementary Fig. S1).

**Table 1:** Classification model performance for GL261 in different tumor regions.

	All	95% CI	Model Performance Evaluation: GL261					95% CI
			Center	95% CI	Middle	95% CI	Edge	
AUC	0.923	—	0.909	—	0.960	—	0.732	—
Accuracy	0.908	0.904, 0.911	0.840	0.837, 0.843	0.87	0.861, 0.875,	0.72	0.72, 0.73
Specificity	0.92	0.91, 0.92	0.811	0.808, 0.815	0.919	0.910, 0.928	0.69	0.68, 0.70
Sensitivity	0.893	0.889, 0.90	0.88	0.87, 0.88	0.786	0.779, 0.793	0.77	0.76, 0.78
F-Score	0.888	0.884, 0.891	0.824	0.821, 0.827	0.821	0.812, 0.829	0.69	0.69, 0.70

**Table 2:** Classification model performance for U87 in different tumor regions.

	All	95% CI	Model Performance Evaluation: U87					95% CI
			Center	95% CI	Middle	95% CI	Edge	
AUC	0.879	—	0.842	—	0.813	—	0.708	—
Accuracy	0.892	0.890, 0.894	0.841	0.839, 0.844	0.779	0.775, 0.782	0.71	0.71, 0.72
Specificity	0.909	0.907, 0.911	0.910	0.908, 0.912	0.768	0.765, 0.772	0.85	0.84, 0.86
Sensitivity	0.85	0.84, 0.86	0.66	0.65, 0.67	0.80	0.78, 0.80,	0.34	0.33, 0.36
F-Score	0.81	0.80, 0.82	0.69	0.68, 0.70	0.73	0.72, 0.74	0.40	0.38, 0.41

**Table 3:** Classification model performance for Daoy in different tumor regions.

	All	95% CI	Model Performance Evaluation: Daoy					95% CI
			Center	95% CI	Middle	95% CI	Edge	
AUC	0.848	—	0.775	—	0.865	—	0.565	—
Accuracy	0.861	0.858, 0.863	0.781	0.777, 0.784	0.79	0.77, 0.79	0.64	0.63, 0.65
Specificity	0.919	0.916, 0.923	0.86	0.86, 0.87	0.88	0.88, 0.89	0.75	0.74, 0.76
Sensitivity	0.73	0.73, 0.74	0.59	0.58, 0.59	0.48	0.46, 0.49	0.40	0.39, 0.42
F-Score	0.77	0.76, 0.77	0.62	0.61, 0.62	0.51	0.49, 0.53	0.42	0.40, 0.43

**Figure 3:**
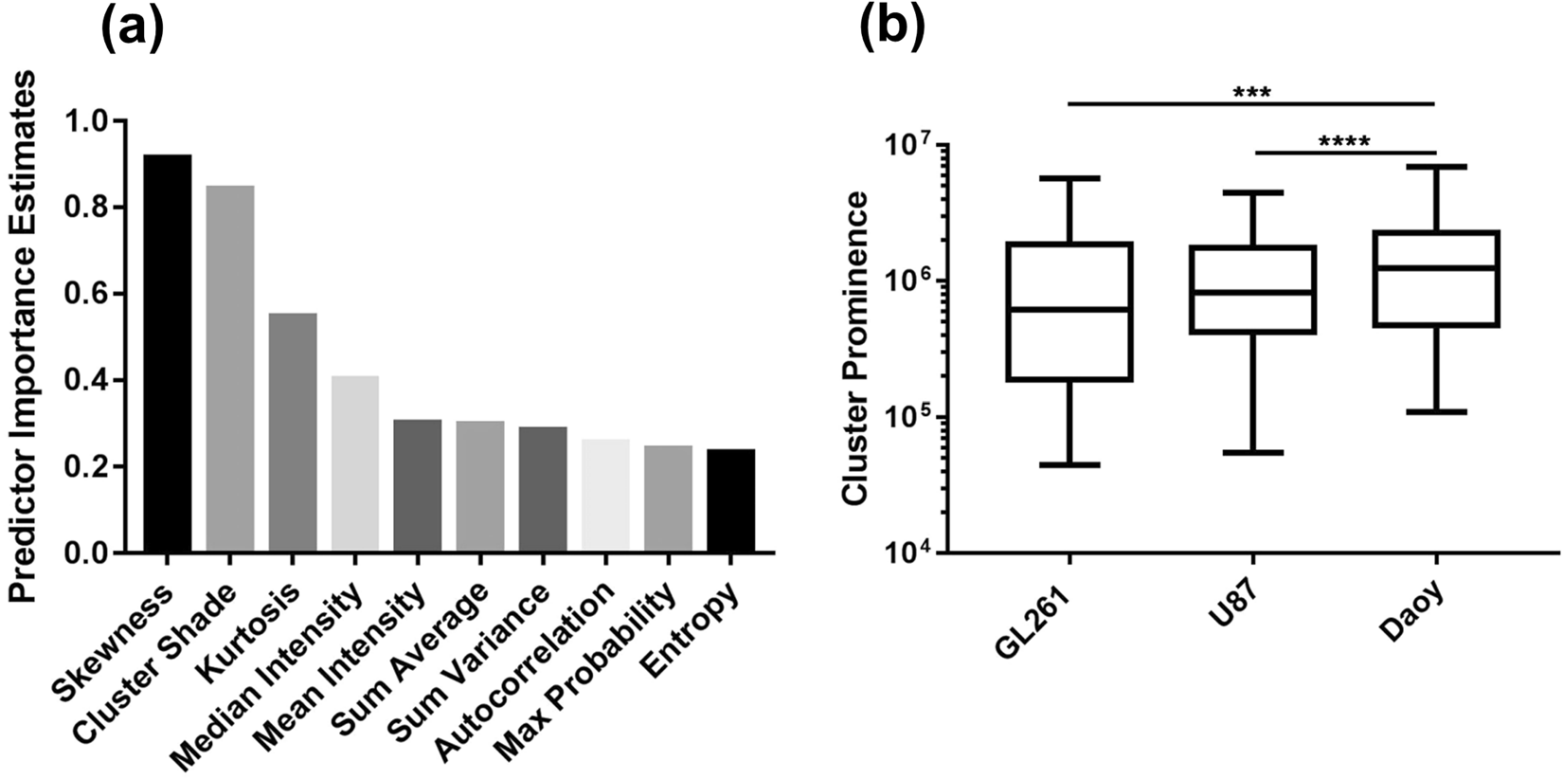
Importance of features in tumor classification. (**a**) Feature importance was estimated for the random forest model using the entire tumor volume and a GLCM of size 512. The top 10 most important features included both 1st and 2nd order texture features. (**b**) Cluster prominence derived from a GLCM of size 512 was calculated for each tumor type. Cluster prominence was significantly difference between medulloblastoma and glioma, both U87 and GL261 tumors. Box plot represents 25th and 75th percentiles; bar = min and max values; line = median, ***p < 0.0002, ****p < 0.0001.

Random forest is an ensemble learning technique which grows many decision trees where the final class is determined by a majority “vote” from all the decision trees. Analysis of the estimated feature importance reveals skewness (0.91) to be the most important feature followed by cluster shade (0.84), kurtosis (0.55), median intensity (0.40), mean intensity (0.30), sum average (0.30), sum variance (0.289), autocorrelation (0.26), max probability (0.24) and entropy (0.23), where 0 represents the smallest possible importance and higher values have greater importance (Fig. 3).

Direct comparison of the image features derived from the GLCM with 512 gray levels showed cluster prominence to be the only feature that differentiates glioma tumors from medulloblastoma. Cluster prominence, which measures the asymmetry of the GLCM, was significantly lower in both glioma models (p ≤ 0.0001 to U87 and GL261) compared to medulloblastoma (GL261 = (1.3 ± 1.7) × 10^6^, U87 = (1.2 ± 0.6) × 10^6^, Daoy = (1.6 ± 1.1) × 10^6^) (Fig. 3). This indicates that gliomas have a lower local variance in gray levels.

### Growth curve prediction

**Figure 4:**
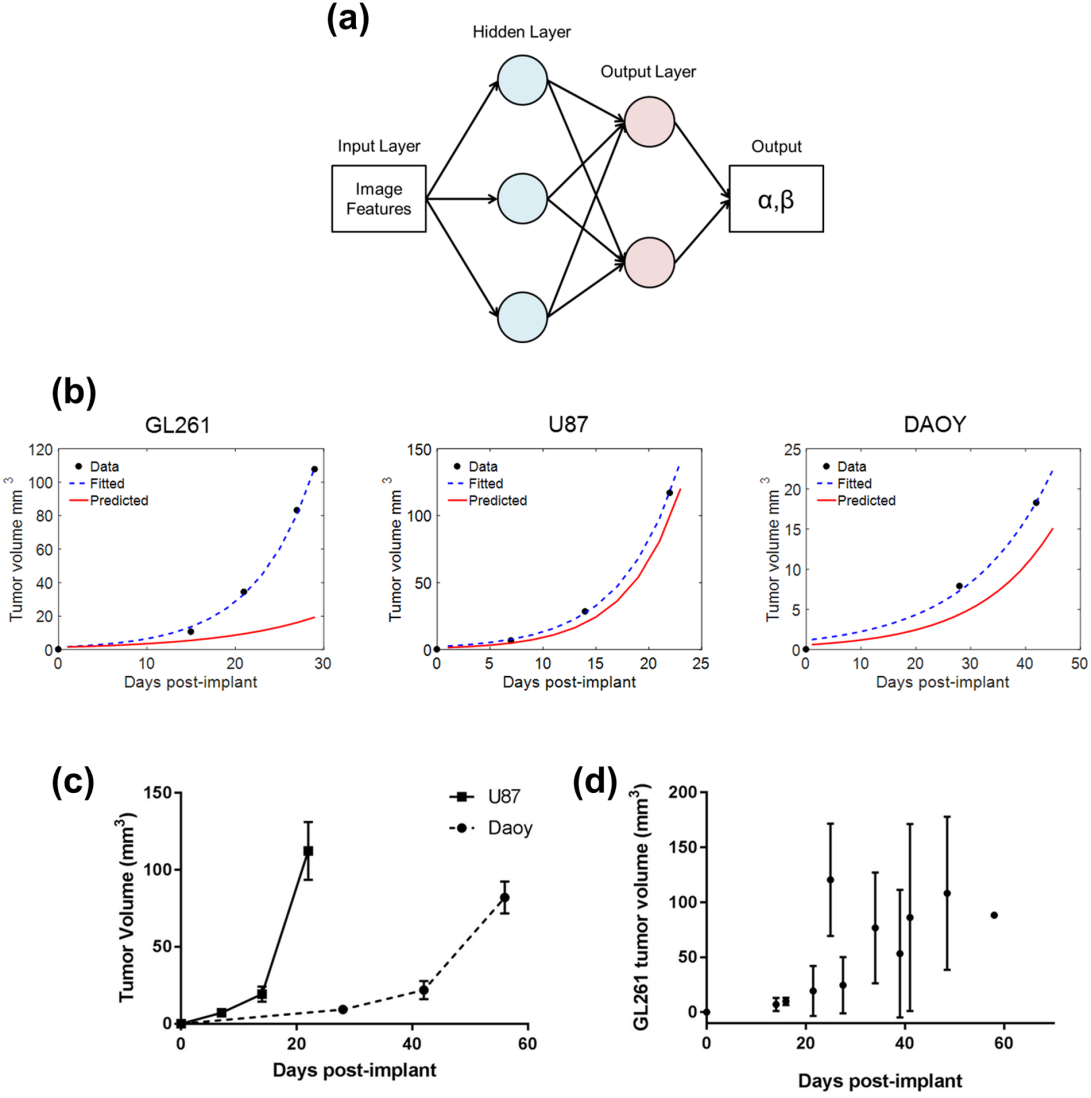
Prediction of tumor growth curve. (**a**) Two-layer feedforward network with a sigmoid transfer function in the hidden layer and linear transfer function in the output layer. Tumor growth is different between the different tumor types. (**b**) Growth curve of the three different tumor type with the experimental data, the fitted exponential growth model and the predicted growth curve from the neural network. (**c**,**d**) Growth curve of each tumor type. Data is shown as mean ± standard deviation.

Using a shallow feedforward two layer neural network, the tumor growth curve could be established with mean square error of 16% (Fig. 4a). The neural network was trained to predict the αβ value for an exponential growth model. The inputs used to train the network were image features from similar tumor volumes and the αβ value from the fitted exponential of the experimental data (Fig. 4b). The experimental data showed U87 to be highly aggressive with a rapid growth rate (α = 1.2 ± 0.8, β = 0.21 ± 0.03) with Daoy having a slower growth rate (α = 0.9 ± 0.5, β = 0.08 ± 0.04) (Fig. 4c). GL261 tumors had higher variance in tumor growth compared to other tumor types (α = 0.7 ± 0.9, β = 0.15 ± 0.04) (Fig. 4d). Overall, the neural network was able to predict tumor growth more accurately for U87 and Daoy tumors than GL261. Additional results can be found in Supplementary Fig. S2.

## Discussion

In this study we have demonstrated that by using texture analysis, standard medical images can be used to classify tumors and predict tumor growth rate in preclinical models. Furthermore we have shown that the size of the GLCM or number of gray levels impacts the performance of the classification models and have identified a classifier that outperformed all others. The best performing classifier was a random forest model with texture features derived from the 512 gray level GLCM that used the entire tumor volume. These derived texture features can be used to predict tumor growth using a shallow two layer neural network. Assuming an exponential growth curve, the neural network is able to predict the αβ value using only the diagnostic scan. These classification and prediction models can provide an additional tool for tumor diagnosis and could be used to better personalized treatment planning without additional burden to the patient.

Previous works have shown that image features can be used for brain tumor classification and grading^32–35^. Studies aimed at using images to classify tumor type used different classification models and parameters and different scan sequences with varying degrees of success (77 to 91% accuracy)^32,34,36^. Our results of 84% accuracy is comparable to these other models by optimizing the GLCM size and classifier. Taking a more reductionist approach, our study focused on the robustness of texture features for different GLCM sizes and how this in turn affects the performance of different classifiers’ while controlling for any external confounding factors such as machine variance by using our preclinical tumor models. Changing the distance between neighbors to construct the GLCM is another variable that can be optimized. However our preclinical tumor volume is limited, therefore extending the distance between neighboring pixels would not produce meaningful results. However, Chen *et al*. has shown in texture analysis of breast lesions that extending the distance of neighbors does not significantly affect the GLCM, a finding which may be applicable to brain tumor studies^37^. We have identified four features (autocorrelation, cluster prominence, sum of squares, and sum variance) that were dependent on the number of gray levels while the other texture features were independent. Cluster prominence, which is a measure of the GLCM asymmetry, was the only feature that was significantly lower in the glioma (GL261 and U87) tumors than medulloblastoma. The observed lower local variance, as defined by cluster prominence, in the glioma tumor model can be attributed to the well-defined tumor border in gliomas unlike the diffuse borders of medulloblastomas (Fig. 1). This finding agrees partially with Brynolfsson’s *et al*. work using apparent diffusion coefficient (ADC) maps of glioma and prostate data^38^. However, this study identified more features being influenced by GLCM size and noted a greater effect of GLCM size on the texture feature value^38^. This seems to indicate that the influence of the GLCM size on texture feature value may vary between image acquisition protocols. Our results indicate that GLCM size is an important parameter to consider when constructing classification models. Therefore to have meaningful comparison across acquisition protocols, the GLCM size should be standardized and not part of the optimization process.

Classifier performance was impacted by the GLCM size, but the selection of the ROI for the derivation of the GLCM had the greatest impact on performance. Using the entire tumor volume resulted in higher accuracy than using a single image slice alone. This may be due to an increase in counting statistics and less sparse GLCMs which allows for extraction of meaningful features. This was the case at the edge of tumors where the ROI was small which resulted in poor performance for all three classifiers. Furthermore, using the entire tumor region would better approximate the tumor’s heterogeneity.

To our knowledge, this is the first demonstration of the use of texture features to predict tumor growth using a neural network. While there are many classical models of tumor growth, we chose to fit the experimental data to an exponential growth curve since the model is straightforward (with only two parameters) and provides a good fit to our data^39^. More complex mathematical models of tumor growth hasve also been proposed which are driven by the tumor’s biological processes however the majority of these models are derived and optimized for gliomas^40–42^. Using a simple two-parameter growth model with minimal assumptions about the underlying tumor biology allows us to test the predictive power of image features with different tumor types. Overall the neural network is able to predict the αβ with better performance for U87 and Daoy tumors with 16% mean square error. The prediction model was best for tumors that had lower growth variance which helps reinforce the network. The high variance in the GL261, a mouse glioma cell line, tumor growth curve may be due to immunological factors in the animal model (Fig. 4). In the GL261 model, cells are implanted into an immunocompetent animal, unlike the human U87 or Daoy cells, potentially leading to an immune response that could delay tumor growth to varying degrees.

While there are other image features that can be used, in this study we are limited to features derived from the signal intensity histogram (first order) and features derived from the GLCM (second order). Differences in image processing between our in-house MATLAB program and other publicly available software tools means that the extracted features’ values are not necessarily identical or universally comparable even though the same mathematical equations were used^29–31^. As such, the use of image features from other software can impact the performance of the classifiers we developed. The classifiers were also trained on scans that were acquired from the same animals at different time points with the assumption that these scans are not necessarily equivalent. This assumption allowed the classification models to be constructed with the use of large cohorts; however this assumption should be investigated in future studies. Another limitation of our work is the use of preclinical brain tumor models that prevents the findings from this study from being directly extrapolated to patient data. Since we are using supervised machine learning algorithms, the classifier relies heavily on the training dataset. Preclinical models can generate these datasets in a timely manner while controlling for confounding factors such as acquisition and reconstruction protocols which are not always readily available in clinical data sets. While there are datasets available for adult gliomas including glioblastoma and low grade glioma^43,44^ and multi- institutional efforts to manually annotate data from The Cancer Imaging Archive (TCIA)^45^, to our knowledge, there are no publically available imaging datasets, at this time, for medulloblastomas. As datasets become available, the established workflow can be implemented to test clinical data. Furthermore, with a preclinical model, we can track tumor growth and establish the growth curve which is not possible with patient data. This work provides proof-of-concept that texture features can classify tumor types with high accuracy and when interpreted by neural networks, these features can map out tumor growth.

These findings demonstrate that image features extracted from standard medical images have the ability to make diagnoses and even predict tumor growth rate. For patients who are not eligible for a biopsy or tumor resection, with further validation using clinical data, this modeling can be an alternate source of information to help clinicians make better informed treatment plans. Furthermore, for patients who may not be immediately eligible for treatments such as radiation therapy, mapping out the growth curve of these tumors can help clinicians identify critical time points when planning the course of treatment. More importantly, this study adds to the previous body of work on the impact of GLCM size on texture feature value.

## Conclusion

Features derived from standard-of-care images can be used to classify tumor type and map tumor growth using machine learning algorithms. The number of gray levels in the construction of the GLCM influenced the performance of the predictive models. A GLCM size of 512 with a random forest classification model yielded an accuracy of 84% based on a tenfold cross-validation in our preclinical glioma and medulloblastoma tumor models. These results are promising in achieving a noninvasive marker for tumor type classification. These texture features are also found to be informative of tumor growth. Using a two layer neural network, the αβ values were predicted from the image features. The neural network had mean squared error of 16.02% with better performance for U87 and Daoy tumors compared to GL261 tumors. The performance of these models can be greatly improved with the addition of new data sets since both the random forest and neural network relied heavily on training data sets. Finally, standardization of feature extraction and exploration of deep learning techniques can contribute to a more accurate prediction of tumor type and growth curve, including a standardized GLCM that can allow for meaningful comparison of texture features.

## Method

### Tumor implantation

All animal studies were approved and performed in accordance with guidelines established by Baylor College of Medicine Institutional Animal Care and Use Committees (IACUC). Severe combined immunodeficient mice (Jackson Lab, Bar Harbor, ME) were implanted in the cerebellum with 1 × 10^6^ Daoy cells (ATCC, Manassas, VA) suspended in matrigel (Sigma-Aldrich, St. Louis, MO) to establish the medulloblastoma tumor model (n = 10). Glioma models were established in the caudate putamen. The human glioma model was established in severe combined immunodeficient mice with 1 × 10^6^ U87 (ATCC, Manassas, VA) cells suspended in matrigel (n = 8). The mouse glioma model was established in C57BL/6 Albino with 5 × 10^5^ GL261 (ATCC, Manassas, VA) cells in matrigel (n = 17). Animals were monitored for general health and euthanized according to the established protocol.

### Texture feature extraction

Animals were imaged either weekly or biweekly based on tumor type, with T_1_-weighted relaxation time = 1500 ms, echo time = 8.5 ms, matrix size 256 × 256, pixel spacing 0.117 × 0.117 mm, slice thickness 0.5 mm) post contrast scan on a 9.4 T magnet (Bruker BioSpin). Gadolinium (Magnevist^®^, 0.1 µL/g diluted in sterile saline) was administered intravenously 10 minutes prior to start of image acquisition. The acquired axial scans (n = 87 scans) were used for texture feature extraction. Due to tumor burden, not all animals were imaged the same number of times.

Image features were extracted using a custom program developed in the 2016 MATLAB program (The MathWorks Inc., Natick, MA). The program allows the users to import the image files and manually select a region of interest (ROI) using a graphical user interface (GUI). The GUI provides visualization of the image and the user segments the ROI by manually defining the perimeter of the tumor. Tumor region segmentation was performed on the central, middle, edge and entire tumor region. The central slice was defined as the slice with the largest cross section (n = 73); the edge of the tumor was designated as the second to last slice where the tumor was visible (n = 69); and the middle tumor region was defined as halfway between the center and tumor edge (n = 34). The middle and edge slices were random slices selected from either side of the tumor. If the tumor was too small, we did not include the edge and/or middle tumor region in the analysis hence there were differences between the number of slices analyzed for each tumor region. For the GLCM representing the entire tumor region, the texture features were calculated by averaging the GLCM from each image slice. Tumor segmentation was manually performed by selecting the tumor border as delineated by the enhancement from the imaging contrast agent. The GLCM was constructed using MATLAB’s built-in *graycomatrix* function which creates a GLCM from an image with the specified number of gray levels, offset. In this study the segmented tumor region gray level intensity binning is performed in the entire image.

Once the ROI or tumor region was segmented 33 different image features are automatically extracted as defined by Harlick *et al*. with the corresponding unique code provided by the international Image Biomarker Standardization Initiative (IBSI)^23,46^ (see Supplementary Table S1). These image features include both first and second order features. Second order features were derived from the gray level co-occurrence matrix (GLCM). The GLCM is a N × N matrix which represents the frequency in which combination of grayscale intensity occurs. The GLCM is defined as for a given matrix size N:

1 $${\boldsymbol{P}}({\boldsymbol{i}},{\boldsymbol{j}};\,{\boldsymbol{\delta }},\,{\boldsymbol{\alpha }})$$

Where N is defined by the number of discrete gray level intensities, each (*i*,*j*) element represent the frequency in which the combination of intensity level i and j occur as separated by pixel distance (δ) in direction (α).

First order features were derived from the grayscale intensity distribution histogram of the pixels from the selected ROI (tumor region). Second order image features were derived from the GLCM constructed with 10 different dimensions (N = 8, 16, 24, 32, 48, 64, 98, 128, 256, 512) which included features from Haralick *et al*., Soh *et al*. and Clausi *et al*.^23,47,48^. The final texture features were extracted from the normalized GLCM by averaging the four different offsets (α = 0°, 45°, 90° and 135° with symmetry and pixel distance of δ = 1). The list of image features can be found in Supplementary Table S1.

### Tumor type classification model

The classifications models were constructed in MATLAB with three classes: GL261, U87 and Daoy and the image features as inputs. In this study, three different classification models were investigated; decision tree, random forest, and support vector machine. Each model used the extracted image features as inputs to predict the three tumor classes or tumor type. Decision tree models were constructed using the *fitctree* function in MATLAB which fits binary decision trees for multiclass classification, with the default setting and split criterion set to Gini’s diversity index. Hyperparameters were optimized to minimize the cross-validation error for all eligible parameters which included max number of splits and minimum number of observation at each node. Random forest models were constructed using the *TreeBagger* function in MATLAB which grows the decision tree by bootstrapping samples of the dataset and selecting a random subset of predictors to use at each split. Default settings were used with the exception of using an ensemble of 500 decision trees. Support vector machine models were constructed using the *fitcecoc* function in MATLAB which produces a multiclass support vector machine model using a Gaussian kernel function. The models were trained using default settings and hyperparameters were optimized to minimize the cross-validation error. The following parameters were optimized:, box constrainedt, penalty imposed on samples for outside of margins), kernel scale, polynomial kernel function order to compute the Gram matrix and the standardization of the input features. To evaluate each algorithm’s performance and prevent overfitting during the training phase, a 10-fold cross validation was performed, where 90% of the data is randomly sampled for training and 10% withheld for testing. The training and testing dataset is partitioned based on individual imaging scans and not by animal. All hyperparameters were optimized using Bayesian optimization. Feature importance for random forest was determined by the summation of changes in error due to node removal and normalizing by the number of branching points.

### Tumor growth rate prediction

Tumor growth curves from each individual tumor were first fitted to a one-term exponential:

2 $$tumor\,volume=\alpha {e}^{\beta \ast time}$$

The αβ values were fitted using the Trust-Region algorithm with default setting using MATLAB for each tumor. The values found from the fit was used as the target value for the neural network with image features derived from early scans (first imaging session) used as input. The two layer neural network used consistied of 33 hidden neurons with a sigmoid transfer function in the hidden layer and linear transfer function in the output. The network was trained with Levenberg-Marquardt back-propagation algorithm where 60% of the data was used for training, 35% used for validation and 5% used for testing (n = 6, 2 test cases for each tumor type from separate cohort). Training and testing samples were divided to have similar distribution and equal representation of all three tumor types.

### Statistical analysis and model performance evaluation

Statistical analysis was performed using GraphPad Prism (GraphPad Software, La Jolla, CA). Model performance was assessed with the following metrics: accuracy, specificity, sensitivity and F-score. TP = true positive, TN = true negative, FP = false positive, FN = false negative:

3 $$Accuracy=\frac{TP+TN}{TP+FP+FN+TN}$$

4 $$Specificity=\frac{TN}{FP+TN}$$

5 $$Sensitivity=\frac{TP}{TP+FN}$$

6 $$Fscore=\frac{2\ast TP}{2\ast TP+FP+FN}$$

The metrics were computed for each individual tumor class from the confusion matrix. Analysis of variance (ANOVA) was used to compare multiple means for each image feature between different tumor types. Bonferroni correction was used for multiple comparisons with P-values presented as multiplicity adjusted p-values with alpha set to 0.05, threshold for significance = 0.000505051, to determine statistical significance of the image features. This stringent correction accounts for 99 potential hypotheses for the three tumor types and thirty-three possible image-based features. P-values were not additionally adjusted for GLCM size, which was set during the choice of optimal model.

## Supplementary information

Supplementary info

## Data Availability

The datasets generated and/or analyzed during the current study are available on https://github.com/tien-tang/tumor-classification_growth-rate.
