# Supplementary material for: Image-based Classification of Tumor Type and Growth Rate using Machine Learning: a preclinical study
Source: Sci Rep. 2019 Aug 29;9:12529. doi: 10.1038/s41598-019-48738-5 (PMC6715812; doi:10.1038/s41598-019-48738-5)
Supplement: Supplementary file 1 — Supplementary info [file 41598_2019_48738_MOESM1_ESM.docx]

**Image-based Classification of Tumor Type and Growth Rate using Machine Learning: a preclinical study**

Tien T. Tang^ab^, Janice A. Zawaski*^b^, Kathleen N. Francis^a^, Amina A. Qutub^a^,

and M. Waleed Gaber*^a,b^

^a^Department of Bioengineering, Rice University, 6500 Main Street, Suite 1030, Houston, TX 77030, USA

^b^Department of Pediatrics, Hematology-Oncology Section, Dan L. Duncan Cancer Center, Baylor College of Medicine, 1102 Bates Street, Suite 200, Houston, TX 77030, USA

*Corresponding authors

**Supplementary Figure S1**

**
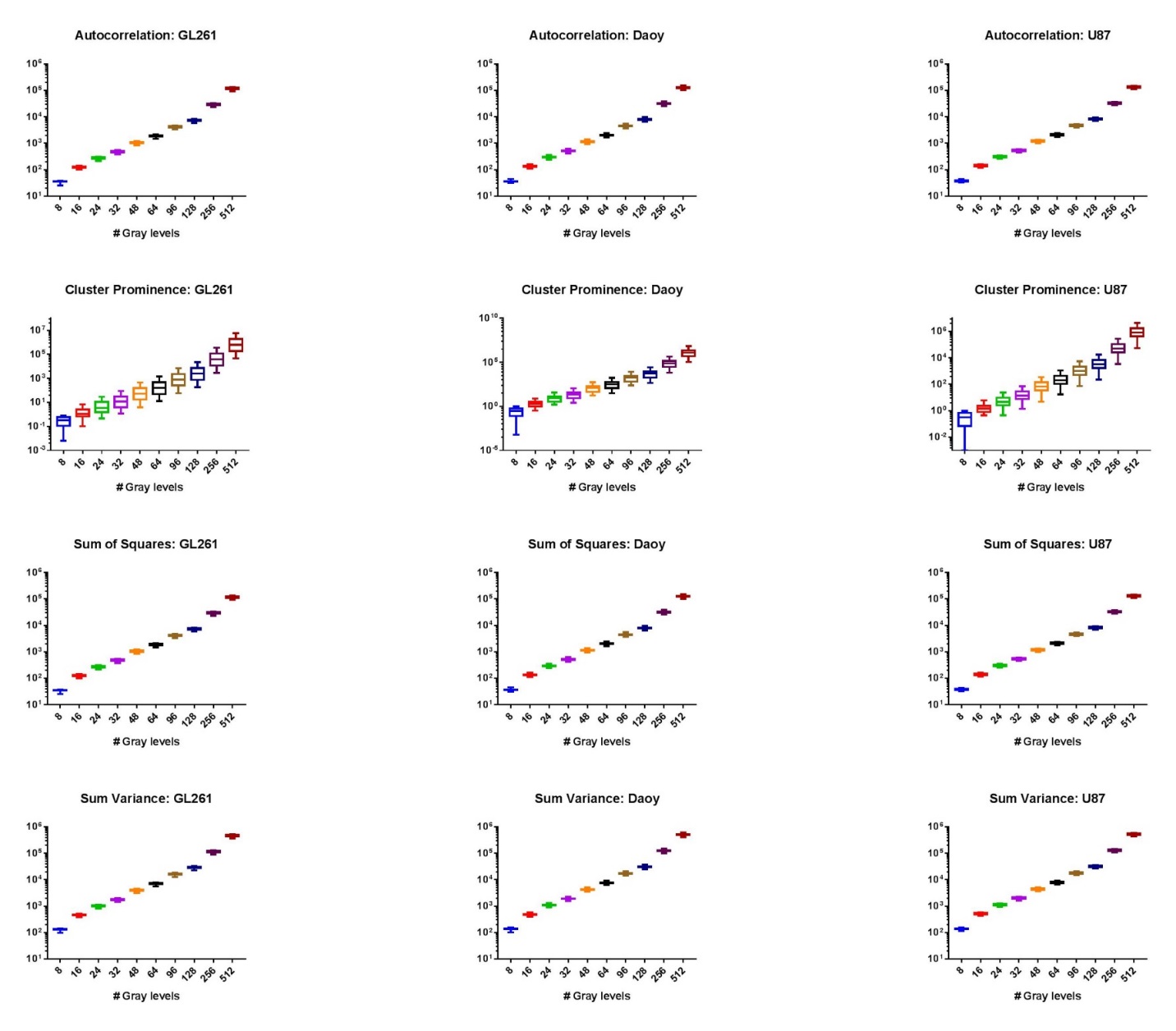
**

**Supplementary Figure S2**

**
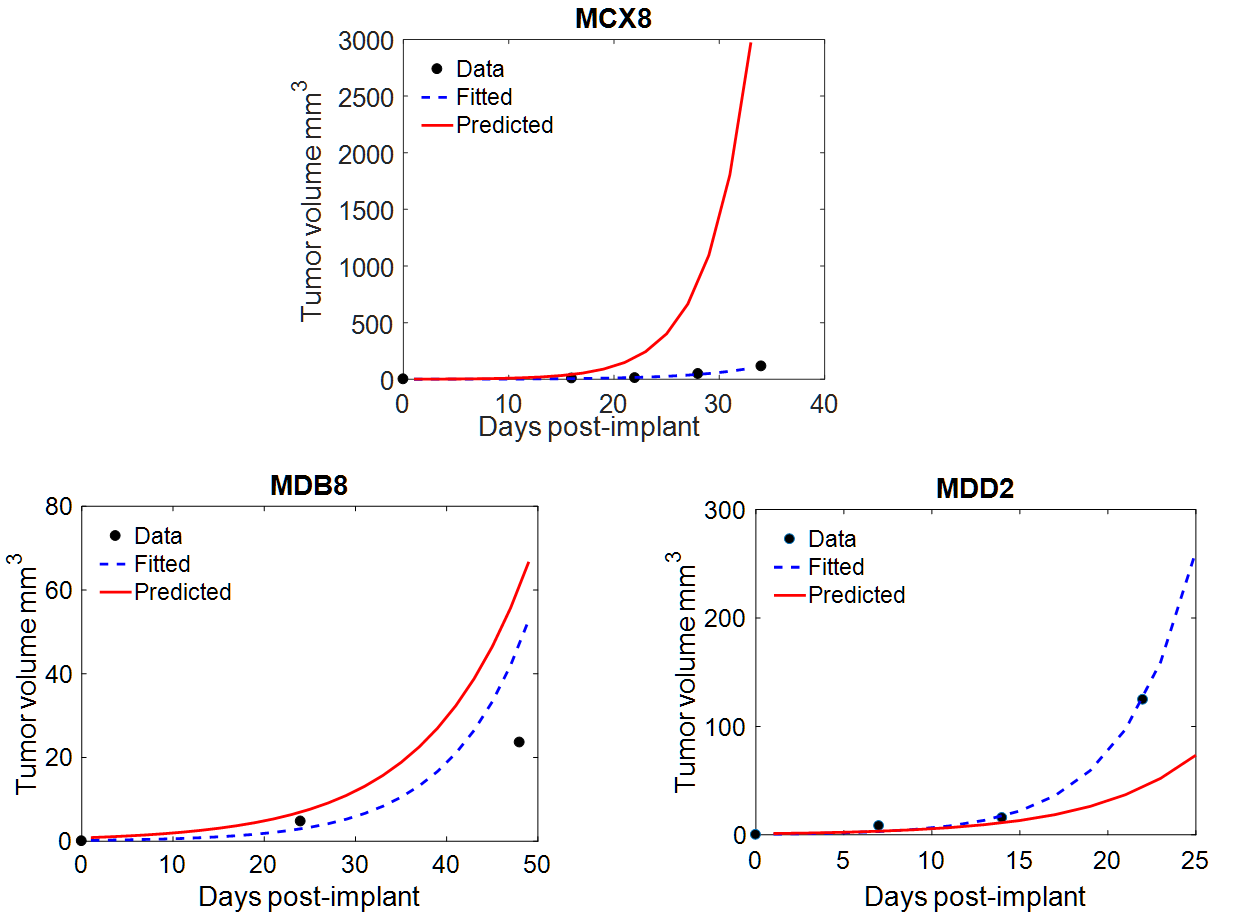
**

**Supplementary Table S1**

| **Order of Features** | **Derived from:** | **Features** | **IBSI Code**[**^1^**](#_ENREF_1) |
| --- | --- | --- | --- |
| First | Pixel Intensity Histogram | Area  Kurtosis  Max  Mean  Median  Min  Range  Skewness  Standard deviation  Variance  Zernike | -  C3I7  3NCY  X6K6  WIFQ 1PR8  5Z3W 88K1  -  CH89  - |
| Second | Grey Level Co-occurrence Matrices (GLCM) | Autocorrelation  Cluster Prominence  Cluster Shade  Contrast  Correlation 1  Correlation 2  Difference Entropy  Difference Variance  Dissimilarity  Energy  Entropy  Homogeneity 1  Homogeneity 2  Information Measure of Correlation 1  Information Measure of Correlation 2  Inverse Difference Normalized  Maximum Probability  Inverse Moment Normalized  Sum Average  Sum Entropy  Sum of Squares: Variance  Sum Variance | QWB0  AE86  7NFM  ACUI  -  NI2N  NTRS  D3YU  8S9J -  TU9B -  -  R8DG  JN9H  NDRX  GYBY  1QCO  ZGXS  P6QZ  UR99  OEEB |

**Supplement Table 2-4**

| Tumor region used for classification model  (Validation Accuracy±Standard Deviation) – Decision Tree | | | | |
| --- | --- | --- | --- | --- |
| # Gray levels | **All** | **Center** | **Middle** | **Edge** |
| 8 | 0.69 ± 0.03 | 0.76 ± 0.02 | 0.74 ± 0.04 | 0.45 ± 0.02 |
| 16 | 0.72 ± 0.02 | 0.776 ± 0.013 | 0.764 ± 0.008 | 0.46 ± 0.04 |
| 24 | 0.73 ± 0.03 | 0.73 ± 0.02 | 0.73 ± 0.02 | 0.46 ± 0.04 |
| 32 | 0.81 ± 0.02 | 0.767 ± 0.016 | 0.75 ± 0.02 | 0.56 ± 0.05 |
| 48 | 0.81 ± 0.02 | 0.72 ± 0.03 | 0.731 ± 0.019 | 0.571 ± 0.015 |
| 64 | 0.82 ± 0.02 | 0.71 ± 0.03 | 0.733 ± 0.015 | 0.51 ± 0.03 |
| 96 | 0.82 ± 0.02 | 0.71 ± 0.03 | 0.75 ± 0.02 | 0.5 ± 0.03 |
| 128 | 0.80 ± 0.02 | 0.67 ± 0.04 | 0.707 ± 0.018 | 0.55 ± 0.007 |
| 256 | 0.82 ± 0.02 | 0.72 ± 0.03 | 0.68 ± 0.03 | 0.49 ± 0.05 |
| 512 | 0.80 ± 0.02 | 0.74 ± 0.03 | 0.751 ± 0.018 | 0.46 ± 0.03 |

| Tumor region used for classification model  (Validation Accuracy±Standard Deviation) – Random Forest | | | | |
| --- | --- | --- | --- | --- |
| # Gray levels | **All** | **Center** | **Middle** | **Edge** |
| 8 | 0.789 ± 0.03 | 0.726±0.03 | 0.647±0.05 | 0.435±0.04 |
| 16 | 0.737 ± 0.02 | 0.753±0.03 | 0.676±0.04 | 0.450±0.04 |
| 24 | 0.776 ± 0.02 | 0.685±0.03 | 0.735±0.03 | 0.507±0.04 |
| 32 | 0.816 ± 0.02 | 0.740±0.03 | 0.706±0.03 | 0.435±0.04 |
| 48 | 0.803 ± 0.02 | 0.671±0.03 | 0.676±0.03 | 0.478±0.04 |
| 64 | 0.789 ± 0.03 | 0.712±0.03 | 0.706±0.04 | 0.450±0.04 |
| 96 | 0.776 ± 0.02 | 0.671±0.03 | 0.706±0.03 | 0.536±0.04 |
| 128 | 0.816 ± 0.03 | 0.753±0.03 | 0.735±0.04 | 0.493±0.04 |
| 256 | 0.842 ± 0.02 | 0.726±0.02 | 0.765±0.04 | 0.565±0.04 |
| 512 | 0.842 ± 0..03 | 0.740±0.02 | 0.735±0.03 | 0.522±0.04 |

| Tumor region used for classification model  (Validation Accuracy±Standard Deviation) – Support Vector Machine | | | | |
| --- | --- | --- | --- | --- |
| # Gray levels | **All** | **Center** | **Middle** | **Edge** |
| 8 | 0.83 ± 0.02 | 0.69 ± 0.02 | 0.73 ± 0.03 | 0.48 ±0.03 |
| 16 | 0.778 ± 0.015 | 0.72 ± 0.02 | 0.79 ± 0.04 | 0.50 ± 0.04 |
| 24 | 0.78 ± 0.02 | 0.72 ± 0.02 | 0.69 ± 0.03 | 0.48 ± 0.03 |
| 32 | 0.794 ± 0.017 | 0.68 ± 0.03 | 0.77 ± 0.04 | 0.50 ± 0.04 |
| 48 | 0.836 ± 0.016 | 0.75 ± 0.02 | 0.76 ± 0.03 | 0.50 ± 0.03 |
| 64 | 0.78 ± 0.02 | 0.71 ± 0.02 | 0.81 ± 0.04 | 0.45 ± 0.04 |
| 96 | 0.80 ± 0.03 | 0.74 ± 0.03 | 0.82 ± 0.04 | 0.54 ± 0.04 |
| 128 | 0.73 ± 0.02 | 0.70 ± 0.017 | 0.81 ± 0.03 | 0.49 ± 0.03 |
| 256 | 0.76 ± 0.02 | 0.76 ± 0.02 | 0.72 ± 0.04 | 0.50 ± 0.04 |
| 512 | 0.841 ± 0.018 | 0.73 ± 0.03 | 0.80 ± 0.02 | 0.46 ± 0.02 |

**Supplement Table 5**

|  | **Fitted (α,β)** | **Predicted (α,β)** |
| --- | --- | --- |
| GL261-MCX3 | 1.461, 0.149 | 1.425, 0.09 |
| GL261-MCX8 | 0.446, 0.163 | 0.775, 0.25 |
| Daoy-MDB2 | 1.167, 0.0657 | 0.577, 0.0726 |
| Daoy-MDB8 | 0.185, 0.115 | 0.795, 0.0904 |
| U87-MDD8 | 2.140, 0.182 | 1.215, 0.120 |
| U87-MDD2 | 0.540, 0.2473 | 1.009, 0.171 |

1 Zwanenburg, A., Leger, S., Vallières, M. & Löck, S. Image biomarker standardisation initiative. *arXiv preprint arXiv:1612.07003* (2016).
